# 1,5-Dicyano­anthraquinone

**DOI:** 10.1107/S1600536810007993

**Published:** 2010-03-06

**Authors:** Mahsa Armaghan, Mostafa M. Amini, Seik Weng Ng

**Affiliations:** a Department of Chemistry, General Campus, Shahid Beheshti University, Tehran 1983963113, Iran; b Department of Chemistry, University of Malaya, 50603 Kuala Lumpur, Malaysia

## Abstract

The complete mol­ecule of the title compound, C_16_H_6_N_2_O_2_, which is generated by a crystallographic inversion centre, is almost planar (r.m.s. deviation = 0.04 Å). In the crystal, adjacent mol­ecules are stacked along the *a* axis, with a shortest centroid–centroid separation of 3.826 (2) Å.

## Related literature

For the synthesis, see: Casey *et al.* (1999 ▶); Coulson (1930*a* ▶,*b* ▶). For some applications of anthraquinones, see: Alagesan & Samuelson (1997 ▶); Chang *et al.* (1996 ▶); Cheng *et al.* (1994 ▶); Kuritani *et al.* (1973 ▶); Lin *et al.* (1995 ▶).
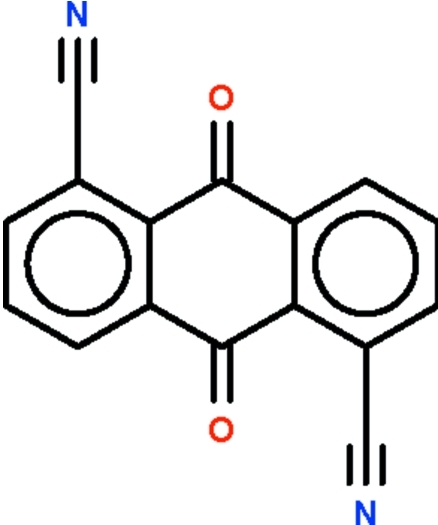


## Experimental

### 

#### Crystal data

C_16_H_6_N_2_O_2_
*M* *_r_* = 258.23
Monoclinic, 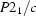

*a* = 3.8256 (10) Å
*b* = 7.0183 (19) Å
*c* = 21.249 (6) Å
β = 91.064 (4)°
*V* = 570.4 (3) Å^3^
*Z* = 2
Mo *K*α radiation
μ = 0.10 mm^−1^
*T* = 293 K
0.35 × 0.06 × 0.03 mm

#### Data collection

Bruker SMART APEX diffractometer
4238 measured reflections
1013 independent reflections
600 reflections with *I* > 2σ(*I*)
*R*_int_ = 0.048

#### Refinement

*R*[*F* ^2^ > 2σ(*F* ^2^)] = 0.053
*wR*(*F* ^2^) = 0.149
*S* = 1.06
1013 reflections
92 parameters
H-atom parameters constrained
Δρ_max_ = 0.19 e Å^−3^
Δρ_min_ = −0.18 e Å^−3^

### 

Data collection: *APEX2* (Bruker, 2009 ▶); cell refinement: *SAINT* (Bruker, 2009 ▶); data reduction: *SAINT*; program(s) used to solve structure: *SHELXS97* (Sheldrick, 2008 ▶); program(s) used to refine structure: *SHELXL97* (Sheldrick, 2008 ▶); molecular graphics: *X-SEED* (Barbour, 2001 ▶); software used to prepare material for publication: *publCIF* (Westrip, 2010 ▶).

## Supplementary Material

Crystal structure: contains datablocks global, I. DOI: 10.1107/S1600536810007993/hb5350sup1.cif

Structure factors: contains datablocks I. DOI: 10.1107/S1600536810007993/hb5350Isup2.hkl

Additional supplementary materials:  crystallographic information; 3D view; checkCIF report

## Figures and Tables

**Fig. 1.**
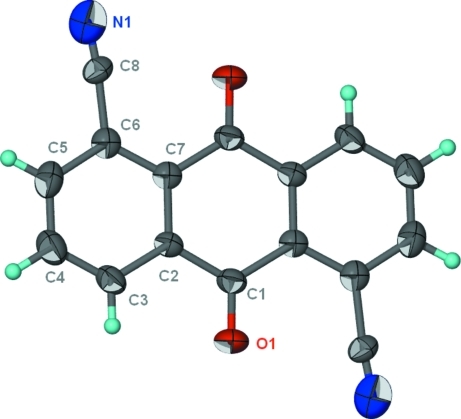
The molecular structure of (I): displacement ellipsoids are drawn at the 50% probability level and H atoms are of arbitrary radius. Unlabelled atoms are generated by the symmetry operation (1–x, 1–y, 1–z).

**Fig. 2.**
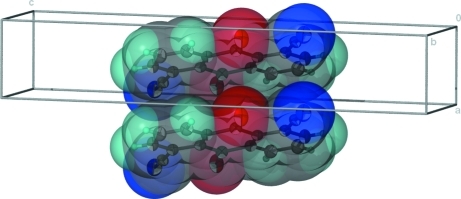
Stacking of the molecules in the unit cell of (I).

## supplementary crystallographic information

### Comment

The title substituted anthraquinone (Scheme I, Fig. 1) was synthesized to study its ability to absorb sulfur from oil when immobilized on silica surface (MCM-41). Anthraquinones are a class of anthracene derivatives having useful industrial applications (Alagesan & Samuelson, 1997; Chang *et al.*, 1996; Cheng *et al.*, 1994; Kuritani *et al.*, 1973; Lin *et al.*, 1995). However, they are usually only sparingly soluble in common oragnic solvents. In the present study, the synthesis involves the exchange of chlorine of 1,5-dichloroanthraquinone with the cyanide of copper cyanide (Coulson, 1930; Casey *et al.*, 1999). The compound is somewhat soluble in DMSO but the recrystallized product is a yellow powder. Crystals were ultimately obtained by diffusing methanol into a DMSO solution of the compound.

The molecule of 1,5-dicyanoanthraquinone, which lies about a center-of-inversion, is planar (max. r.m.s.deviation 0.04 Å). Adjacent molecules are stacked over each other along the *a*-axis of the monoclinic unit cell; the distance is that of the *a*-axial length itself (Fig. 2).

### Experimental

1,5-Dicyanoanthraquinone was prepared by using a reported procedure by reacting 1,5-dichloroanthraquinone with benzyl cyanide in presence of cuprous cyanide (Coulson, 1930a,b; Casey *et al.*, 1999). The compound is sparingly soluble in common solvents; yellow prisms of (I) were obtained by the slow diffusion of methanol into a DMSO solution of the compound; m.p.> 633 K, decompose).

### Refinement

Carbon-bound H-atoms were placed in calculated positions (C—H 0.93 Å) and were included in the refinement in the riding model approximation, with *U*(H) set to 1.2*U*(C).

### Crystal data

**Table:**

C_16_H_6_N_2_O_2_	*F*(000) = 264
*M**_r_* = 258.23	*D*_x_ = 1.503 Mg m^−^^3^
Monoclinic, *P*2_1_/*c*	Mo *K*α radiation, λ = 0.71073 Å
Hall symbol: -P 2ybc	Cell parameters from 614 reflections
*a* = 3.8256 (10) Å	θ = 3.1–25.4°
*b* = 7.0183 (19) Å	µ = 0.10 mm^−^^1^
*c* = 21.249 (6) Å	*T* = 293 K
β = 91.064 (4)°	Prism, yellow
*V* = 570.4 (3) Å^3^	0.35 × 0.06 × 0.03 mm
*Z* = 2	

### Data collection

**Table:**

Bruker SMART APEX diffractometer	600 reflections with *I* > 2σ(*I*)
Radiation source: fine-focus sealed tube	*R*_int_ = 0.048
graphite	θ_max_ = 25.0°, θ_min_ = 1.9°
ω scans	*h* = −4→4
4238 measured reflections	*k* = −8→8
1013 independent reflections	*l* = −25→25

### Refinement

**Table:**

Refinement on *F*^2^	Secondary atom site location: difference Fourier map
Least-squares matrix: full	Hydrogen site location: inferred from neighbouring sites
*R*[*F*^2^ > 2σ(*F*^2^)] = 0.053	H-atom parameters constrained
*wR*(*F*^2^) = 0.149	*w* = 1/[σ^2^(*F*_o_^2^) + (0.0649*P*)^2^ + 0.1468*P*] where *P* = (*F*_o_^2^ + 2*F*_c_^2^)/3
*S* = 1.06	(Δ/σ)_max_ = 0.001
1013 reflections	Δρ_max_ = 0.19 e Å^−^^3^
92 parameters	Δρ_min_ = −0.18 e Å^−^^3^
0 restraints	Extinction correction: *SHELXL97* (Sheldrick, 2008), Fc^*^=kFc[1+0.001xFc^2^λ^3^/sin(2θ)]^-1/4^
Primary atom site location: structure-invariant direct methods	Extinction coefficient: 0.033 (9)

### Fractional atomic coordinates and isotropic or equivalent isotropic displacement parameters (Å^2^)

**Table:**

	*x*	*y*	*z*	*U*_iso_*/*U*_eq_	
O1	0.8448 (7)	0.2052 (3)	0.55023 (10)	0.0691 (8)	
N1	0.0797 (10)	0.7591 (5)	0.30574 (16)	0.0837 (11)	
C1	0.6782 (8)	0.3375 (4)	0.52815 (13)	0.0407 (7)	
C2	0.5829 (7)	0.3390 (4)	0.46013 (12)	0.0373 (7)	
C3	0.6669 (8)	0.1823 (4)	0.42375 (14)	0.0473 (8)	
H3	0.7752	0.0773	0.4423	0.057*	
C4	0.5910 (9)	0.1815 (5)	0.36042 (15)	0.0583 (10)	
H4	0.6438	0.0750	0.3364	0.070*	
C5	0.4369 (9)	0.3378 (5)	0.33232 (15)	0.0566 (9)	
H5	0.3903	0.3369	0.2892	0.068*	
C6	0.3504 (7)	0.4968 (4)	0.36757 (13)	0.0430 (8)	
C7	0.4220 (7)	0.4985 (4)	0.43270 (12)	0.0373 (7)	
C8	0.1889 (8)	0.6593 (5)	0.33253 (14)	0.0424 (8)	

### Atomic displacement parameters (Å^2^)

**Table:**

	*U*^11^	*U*^22^	*U*^33^	*U*^12^	*U*^13^	*U*^23^
O1	0.097 (2)	0.0525 (15)	0.0572 (15)	0.0343 (13)	−0.0103 (13)	0.0056 (11)
N1	0.090 (3)	0.093 (3)	0.068 (2)	−0.006 (2)	0.0044 (19)	−0.0029 (19)
C1	0.0450 (18)	0.0323 (16)	0.0450 (17)	0.0043 (13)	0.0016 (13)	0.0035 (13)
C2	0.0383 (17)	0.0308 (16)	0.0427 (16)	0.0014 (12)	0.0011 (12)	0.0016 (12)
C3	0.053 (2)	0.0347 (18)	0.0540 (19)	0.0052 (13)	0.0015 (14)	−0.0067 (14)
C4	0.063 (2)	0.052 (2)	0.060 (2)	0.0047 (16)	0.0011 (17)	−0.0155 (17)
C5	0.058 (2)	0.070 (2)	0.0414 (17)	−0.0024 (17)	−0.0007 (15)	−0.0078 (16)
C6	0.0400 (17)	0.0455 (18)	0.0437 (17)	−0.0032 (14)	0.0028 (12)	−0.0009 (14)
C7	0.0325 (15)	0.0377 (17)	0.0416 (16)	−0.0027 (12)	0.0024 (11)	0.0024 (12)
C8	0.0388 (18)	0.050 (2)	0.0382 (17)	0.0024 (14)	−0.0053 (13)	0.0059 (15)

### Geometric parameters (Å, °)

**Table:**

O1—C1	1.216 (3)	C4—C5	1.376 (4)
N1—C8	0.991 (4)	C4—H4	0.9300
C1—C7^i^	1.475 (4)	C5—C6	1.387 (4)
C1—C2	1.484 (4)	C5—H5	0.9300
C2—C3	1.386 (4)	C6—C7	1.406 (3)
C2—C7	1.399 (4)	C6—C8	1.490 (4)
C3—C4	1.371 (4)	C7—C1^i^	1.475 (4)
C3—H3	0.9300		
			
O1—C1—C7^i^	121.2 (3)	C5—C4—H4	119.9
O1—C1—C2	119.9 (3)	C4—C5—C6	120.8 (3)
C7^i^—C1—C2	118.8 (2)	C4—C5—H5	119.6
C3—C2—C7	120.5 (3)	C6—C5—H5	119.6
C3—C2—C1	118.8 (2)	C5—C6—C7	119.6 (3)
C7—C2—C1	120.7 (2)	C5—C6—C8	116.5 (3)
C4—C3—C2	120.2 (3)	C7—C6—C8	123.9 (3)
C4—C3—H3	119.9	C2—C7—C6	118.7 (3)
C2—C3—H3	119.9	C2—C7—C1^i^	120.4 (2)
C3—C4—C5	120.2 (3)	C6—C7—C1^i^	120.9 (3)
C3—C4—H4	119.9	N1—C8—C6	174.6 (4)
			
O1—C1—C2—C3	4.2 (4)	C4—C5—C6—C8	179.3 (3)
C7^i^—C1—C2—C3	−177.9 (3)	C3—C2—C7—C6	−0.5 (4)
O1—C1—C2—C7	−173.6 (3)	C1—C2—C7—C6	177.2 (2)
C7^i^—C1—C2—C7	4.3 (4)	C3—C2—C7—C1^i^	177.9 (3)
C7—C2—C3—C4	−0.4 (4)	C1—C2—C7—C1^i^	−4.4 (4)
C1—C2—C3—C4	−178.2 (3)	C5—C6—C7—C2	0.6 (4)
C2—C3—C4—C5	1.3 (5)	C8—C6—C7—C2	−178.4 (3)
C3—C4—C5—C6	−1.2 (5)	C5—C6—C7—C1^i^	−177.8 (3)
C4—C5—C6—C7	0.2 (4)	C8—C6—C7—C1^i^	3.2 (4)

Symmetry codes: (i) −*x*+1, −*y*+1, −*z*+1.
